# Effective Topological Charge Cancelation Mechanism

**DOI:** 10.1038/srep27117

**Published:** 2016-06-01

**Authors:** Luka Mesarec, Wojciech Góźdź, Aleš Iglič, Samo Kralj

**Affiliations:** 1 Laboratory of Biophysics, Faculty of Electrical Engineering, University of Ljubljana, Tržaška 25, 1000 Ljubljana, Slovenia; 2 Institute of Physical Chemistry, Polish Academy of Sciences, Kasprzaka 44/52, 01-224 Warsaw, Poland; 3 Condensed Matter Physics Department, Jožef Stefan Institute, Jamova 39, 1000 Ljubljana, Slovenia; 4 Jožef Stefan International Postgraduate School, Jamova 39, 1000 Ljubljana, Slovenia; 5 Faculty of Natural Sciences and Mathematics, University of Maribor, Koroška 160, 2000 Maribor, Slovenia

## Abstract

Topological defects (TDs) appear almost unavoidably in continuous symmetry breaking phase transitions. The topological origin makes their key features independent of systems’ microscopic details; therefore TDs display many universalities. Because of their strong impact on numerous material properties and their significant role in several technological applications it is of strong interest to find simple and robust mechanisms controlling the positioning and local number of TDs. We present a numerical study of TDs within effectively two dimensional closed soft films exhibiting in-plane orientational ordering. Popular examples of such class of systems are liquid crystalline shells and various biological membranes. We introduce the *Effective Topological Charge Cancellation* mechanism controlling localised positional assembling tendency of TDs and the formation of pairs {*defect, antidefect*} on curved surfaces and/or presence of relevant “impurities” (e.g. nanoparticles). For this purpose, we define an *effective topological charge* Δ*m*_eff_ consisting of *real*, *virtual* and smeared *curvature topological charges* within a surface patch Δ*ς* identified by the typical spatially averaged local Gaussian curvature *K*. We demonstrate a strong tendency enforcing Δ*m*_eff_ → 0 on surfaces composed of Δ*ς* exhibiting significantly different values of spatially averaged *K*. For Δ*m*_eff_ ≠ 0 we estimate a critical depinning threshold to form pairs {*defect, antidefect*} using the electrostatic analogy.

Continuous symmetry breaking phase transitions are ubiquitous and are the main reason behind the rich diversity of patterns observed in nature. In phase transitions topological defects (TDs)^1^ unavoidably appear at least temporarily in a relevant field describing the ordering of the broken phase. Due to topological reasons, physics of TDs features several universalities which are of interest for all branches of physics, spanning particle physics, condensed matter and even cosmology. For example, the first theory of coarsening of TDs following a phase transition quench, was developed in cosmology^2^ to describe TDs in the Higgs field within the early universe. On the other hand, the most experimentally amenable system to study static and dynamic properties of TDs are various liquid crystalline (LC) structures^3^. In addition to fundamental science, the ability to sensitively control and tame TDs is also of high interest in various technological and biomedical applications^4^,^5^,^6^.

Structures of symmetry broken phases are determined by the gauge field^1^,^7^ component of a relevant order parameter pertinent to the phase transition. On the other hand, the degree of established ordering is quantified by its amplitude. If a gauge field is topologically frustrated, topological defects are introduced to intervene between regions imposing competing gauge field structures. The volume enclosing a TD, where the amplitude of the order parameter is substantially suppressed responding to local strong elastic distortions, is referred to as the core^7^,^8^ of the TD. In general, in the core’s centre i) the higher symmetry phase ordering is topologically trapped^9^, and ii) the gauge field is not uniquely defined. The key property of a TD is quantified by the discrete topological charge *q*, which is an additive and a conserved quantity^1^. An isolated topological defect bears *q* ≠ 0 and can not be removed via local continuous transformations. One commonly refers to TDs with a positive and a negative value of *q* as *defect* and *antidefect*, respectively. In general, the presence of TDs is energetically costly and systems tend to avoid them. Consequently, in most cases a nearby pair {*defect*, *antidefect*} mutually annihilates into a locally defectless structure. The Goldstone-type excitations in the gauge field component, which are a direct consequence of broken continuous symmetry, potentially enable an effective long range interaction of TDs with their neighbourhood^7^,^10^. Therefore, gauge fields determine the “sight” of TDs via which they could efficiently probe nearby conditions. On the other hand, controlled variations of gauge fields could be exploited to manipulate the position, assembling and even the number of TDs.

An experimentally adequate and accessible testing bed to analyse structures of TDs in orientational degrees of freedom are effectively two-dimensional soft films possessing in-plane ordering. Among them are of particularly hot recent interest liquid crystalline shells^11^,^12^,^13^,^14^,^15^,^16^,^17^,^18^ and biological membranes^19^,^20^,^21^,^22^. Henceforth, we refer to them as *ordered soft films*. The former systems typically consist of a fluid droplet carrier covered by a thin (few molecular lengths) liquid crystalline film. They are promising candidates for various future photonic applications^11^,^23^. Furthermore, in biological membranes TDs could serve as nucleating sites for diverse biological mechanisms. For example, they could enable anomalous growth or even facilitate cell fission^22^.

These *ordered soft films* could be mathematically treated as an effectively two-dimensional system, where the charge *q* of a TD is equivalent^3^ to its winding number *m*. 2D XY-type minimal models have been commonly used to study TDs in such geometries. Pioneering studies on frozen surfaces^24^,^25^,^26^,^27^ reveal that the Gaussian curvature *K* strongly impacts the position and number of TDs. The Poincaré-Hopff and Gauss-Bonnet theorems^28^ claim that the total topological charge of TDs within a closed surface possessing in-plane order is determined by the total surface integral of *K*. It has been shown^24^,^25^ that electrostatic analogy could be employed to describe the coupling between *K* and *m*. In this analogy *K* and topological charges play the role of an electric field and electric charges^24^,^25^, respectively. It has been demonstrated that surfaces exhibiting regions with both positive and negative *K* could trigger the unbinding^24^,^25^ of pairs of TDs bearing opposite topological charges. The majority of these studies^23^,^24^,^25^,^26^,^27^ were based on a covariant derivative approach, which takes into account only the so called *intrinsic* curvature contributions. In case of in-plane orientational order, these terms penalise departures of the ordering field from surface geodesics. However, latter works by Selinger *et al*.^29^,^30^ and Napoli and Vergori^31^,^32^ demonstrated that the so-called *extrinsic* terms should be also considered and their contribution is reminiscent to an external ordering field. In general, *extrinsic* and *intrinsic* terms might enforce contradicting tendencies^29^,^31^. The *extrinsic* term is also related to a deviatoric term introduced in the study of anisotropic biological membranes and thin shells^20^,^21^,^33^,^34^.

In addition, several recent studies in LCs reveal strong interactions between TDs and nanoparticles (NPs)^6^,^35^,^36^,^37^,^38^,^39^. In case that NPs do not interact with the *nematic director* field (i.e. the gauge field describing the nematic orientational ordering) exhibiting TDs then the NPs often tend to assemble within cores of TDs. However, if a NP strongly interacts with a *nematic director* field, it could effectively act as a topological charge^35^,^18^. In such cases NPs could change the total topological charge of “real” TDs in the system^18^.

In our contribution we study the impact of *K* and NPs, effectively acting as TDs, on the number and position of TDs within the *ordered soft films* exhibiting nematic-type in-plane ordering. We use a Landau-de Gennes-Helfrich type model^40^,^41^,^42^,^43^ formulated in terms of a 2D nematic order tensor **Q** and surface curvature tensor **C.** We introduce the *Effective Topological Charge Cancellation* mechanism exploiting the electrostatic analogy. It predicts assembling tendencies of TDs on surfaces possessing surface patches with significantly different values of *K*. Based on it, we estimate the threshold condition for curvature driven formations of pairs {*defect, antidefect*} on demonstrative geometries.

## Results

We study the nematic ordering on closed, smooth, axially symmetric surfaces. Liquid crystal molecules are bound to lie on the local tangent plane on a surface locally described by the outer unit normal **v**, but are otherwise unconstrained. Local surface curvature is described by the curvature tensor


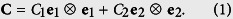


Its unit eigenvectors {**e**_1_, **e**_2_} determine the directions of maximal and minimal curvature, eigenvalues {*C*_1_, *C*_2_} are the corresponding principal curvatures, and **v** = **e**_1_ × **e**_2_. Key invariants formed by **C** are the Gaussian curvature *K* = *C*_1_*C*_2_ and the mean curvature *H* = (*C*_1_ + *C*_2_)/2. Alternatively, the invariant can be also the curvature deviator *D* = (*C*_1_ − *C*_2_)/2^21^,^33^,^34^, where *D*^2^ = *H*^2^ − *K*.

To describe the orientational ordering on a surface, we introduce a surface order tensor **Q**. In the diagonal form it can be expressed as^42^,^43^


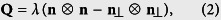


where {**n**, **n**_⊥_} are the eigenvectors of **Q** corresponding to the eigenvalues of {*λ*, −*λ*}, *λ* ∈ [0, 1/2]. The lower bound (*λ* = 0) corresponds to the isotropic state, where the orientational order is lost, while the upper bound (*λ* = 1/2) corresponds to the maximal degree of the orientational order. In general, **n** is classified as a gauge field, and is in LC community commonly referred to as the *nematic director* field. Topological defects are signalled by *λ* = 0.

The total free energy functional of the *ordered soft film* surface *ζ* is expressed as an integral of the sum of the *ordered soft film* isotropic bending energy density (*f*_b_), orientational condensation contribution (*f*_c_), and elastic orientational free energy density (*f*_e_)^42^,^43^:


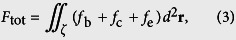


where *d*^2^**r** is an infinitesimal surface element and the integration is carried out over the whole *ordered soft film* surface area *ζ*. The free energy density contributions are expressed as


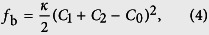



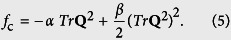



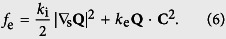


The isotropic bending energy density of a membrane (Eq. (4)) is described within the isotropic spontaneous curvature membrane model^40^,^41^,^44^,^45^,^46^, where *κ* is the membrane bending constant and *C*_0_ is the isotropic membrane spontaneous curvature^34^.

The condensation term enforces the equilibrium nematic ordering amplitude 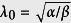, *α* = (*T*_c_ − *T*)*α*_0_, *α*_0_ and *β* stand for positive Landau expansion material dependent coefficients, and *T*_c_ is the phase transition temperature.

The orientational (anisotropic) elastic terms are described by positive *intrinsic* (*k*_i_) and *extrinsic* (*k*_e_) elastic constants, weighting relative importance of *intrinsic* and *extrinsic* (deviatoric) elastic contributions^20^,^21^,^29^,^30^,^31^,^32^,^33^,^34^. The *intrinsic* elastic component is associated with variations of **n** living in 2D curved space. On the other hand, the *extrinsic* elastic component tells how **n** is embedded in 3D space. The difference between these contributions is well visible in an infinitely long cylinder of radius *R*. The *intrinsic* elastic penalty equals to zero for **n**, pointing either along the symmetry axis or at right angles with it. However, the *extrinsic* term is non-zero and it favours **n** to align along the symmetry axis. Details are presented in Supplementary Material. The essential characteristic material dependent length of the model is the order parameter correlation length 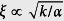, where *k* is an effective representative nematic elastic constant. In cases dominated by the *intrinsic* curvature it holds *k* ~ *k*_*i*_.

In the study we consider either elliptical or dumb-bell shaped closed *ordered soft films* of surfaces *ζ* exhibiting spherical topology. We set *ζ* as surfaces of revolution with rotational symmetry about the *z*–axis within the Cartesian system (*x*, *y*, *z*) defined by the unit vectors triad (**e**_*x*_, **e**_*y*_, **e**_*z*_). Ellipsoidal shapes are parameterised as^42^


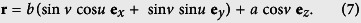


Here, **r** is the position vector of a generic point lying on an ellipsoid surface *ζ*, *v* ∈ [0, *π*] and *u* ∈ [0, 2*π*] stand for zenith and azimuthal angles; 2*a* is the height and 2*b* the width of an ellipsoid. The ellipsoidal films are prolate (oblate) when *η* = *a*/*b* > 1 (*η* < 1). On the other hand, dumb-bell structures are calculated within the spontaneous curvature model. Calculation details are presented in Methods.

### *Effective Topological Charge Cancellation* mechanism

Let us suppose that a closed surface *ζ* consists of surface patches Δ*ζ*, where each patch is characterised by a significantly different average Gaussian curvature 
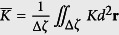
. Within each patch Δ*ζ* we introduce the *effective topological charge*


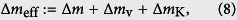


consisting of *real* (Δ*m*), *virtual* (Δ*m*_v_), and smeared *curvature* (Δ*m*_K_) *topological charge*. They are defined as follows: Δ*m* and Δ*m*_v_ represent sums of topological charges of “real” and “virtual” TDs within a patch, respectively. A “real” topological charge displays a singularity in **n** in the centre of TD’s core. On the other hand, a “virtual” TD is formed by an “object” (e.g. a *μm* or a nano-sized particle)^35^ immersed in a LC film, which at the object-LC interface enforces a *nematic director* field resembling a “real” TD as illustrated in Figure S4 in Supplementary Material section. Finally, in spirit of the known fact that local positive (negative) Gaussian curvature acts like a smeared negative (positive) topological charge^24^,^25^, we define Δ*m*_K_ as


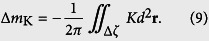


We claim, that within each patch Δ*ζ* there is a “neutralisation” tendency to cancel the *effective topological charge* within it (i.e. to form a local configuration exhibiting Δ*m*_eff_ (Δ*ζ*) = 0), to which we refer as the *Effective Topological Charge Cancellation* (ETCC) mechanism. We term a structure, in which Δ*m*_eff_ (Δ*ζ*) ~ 0 (i.e. Δ*m*_eff_ ≪ *m*_0_) is fulfilled in each patch, the ETCC *limit structure*. Here *m*_0_ represents the smallest unit TD charge, which in nematic LCs equals to *m*_0_ = 1/2. Note, that the *effective topological charge* of any whole closed surface *ζ* equals to zero. Namely, according to the Gauss-Bonnet and Poincaré-Hopff theorems^28^, the total topological charge of an in-plane orientational field is given by


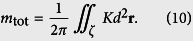


In terms of our definitions it holds 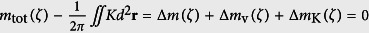, yielding Δ*m*_eff_ (*ζ*) = 0.

In the following paragraphs, we demonstrate the ETCC mechanism neutralisation tendency within patches Δ*ζ* numerically on several examples. In cases Δ*m*_eff_ (Δ*ζ*) ≠ 0 the system i) aims either to globally redistribute existing TDs or ii) exhibits the tendency to form additional pairs {*defect, antidefect*} in order to “neutralise” the *effective topological charge*. We also show that the mechanism enables estimation of threshold conditions to form pairs {*defect, antidefect*}.

### Defect-antidefect depinning threshold

In general, ETCC *limit structures* could be formed either via i) redistribution of TDs or ii) formation of additional TDs obeying the conservation of the total topological charge in the system. In the following paragraphs, we represent key features of the latter case. We analytically estimate the threshold condition for a depinning of pairs {*defect, antidefect*} based on the ETCC mechanism on a relatively simple demonstrative case. In recent reports^24^,^25^,^47^ it has been demonstrated that curvature could trigger depinning for surfaces exhibiting both positive and negative Gaussian curvature. Below, we demonstrate that depinning could be enforced also on surfaces where spatially dependent *K* does not change the sign.

**Figure 1:**
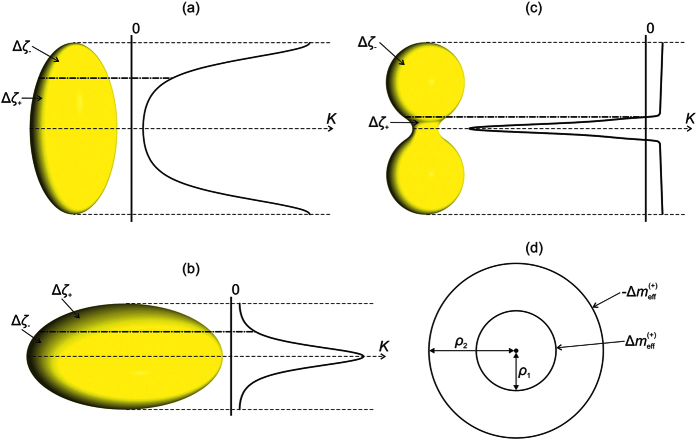
Schematic representation of closed shells and their electrostatic analogue. (**a**) prolate ellipsoid, (**b**) oblate ellipsoid, (**c**) dumb-bell shape. Typical spatial variation of the Gaussian curvature is depicted at the right side of these shapes. (**d**) The electrostatic analogue of structures. The capacitor plates located at *ρ*_1_ and *ρ*_2_ host effective topological charges Δ*m*_eff_ (Δ*ζ*_+_) > 0 and Δ*m*_eff_ (Δ*ζ*_−_) < 0, respectively, where |Δ*m*_eff_ (Δ*ζ*_−_)| = Δ*m*_eff_ (Δ*ζ*_+_).

To illustrate the universality of the mechanism we consider i) surfaces composed of regions exhibiting *K* > 0 and *K* < 0, and also ii) surfaces exhibiting only 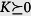. Schematic sketches of (ellipsoidal and dumb-bell) shapes considered in our simulations and corresponding typical spatial variations of *K* are depicted in Fig. 1a–c.

For sake of clarity, in presenting the mechanism we henceforth limit our attention only to structures exhibiting inversion symmetry in the absence of the *extrinsic field*. For symmetry reasons we limit our attention to orientational ordering within upper halves of structures shown in Fig. 1a–c. Within them we introduce two competing surface patches Δ*ζ*_i_ characterised by significantly different values of *K*_i_ = *K* (Δ*ζ*_i_). Here *i* = {+, −} designates the surface patch bearing a {positive, negative} *effective topological charge*. Due to the Poincaré-Hopff and Gauss-Bonnet theorems, it holds Δ*m*_eff_ (Δ*ζ*_+_) + Δ*m*_eff_ (Δ*ζ*_−_) = 0, therefore


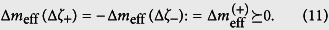


In Fig. 1a–c these competing patches are separated by thick dash-dotted lines, characterised by *K* = *K*_0_. In structures possessing both signs of Gaussian curvature it is natural to set *K*_0_ = 0. For ellipsoids, we set *K*_0_ = 〈*K*〉, where 〈*K*〉 describes the average value of *K* on a closed surface *ζ*. In the latter case, *K*_0_ separates the competing surface patches exhibiting *K* ~ 0 and *K* ≫ 0.

We consider cases where 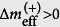 and we employ a two-dimensional electrostatic analogy originally suggested by Bowick *et al*.^24^. Within this analogy a monopole bearing charge *m* creates the *elastic electric field*


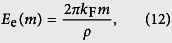


representing the elastic analogue of a 2D electric field, where details are given in the Supplementary Material. Here *k*_F_ is a representative elastic constant and *ρ* stands for the distance from the monopole.

In this view, the upper parts of configurations shown in Fig. 1a–c are roughly analogous to a 2D cylindrical capacitor schematically sketched in Fig. 1d. The two concentric capacitor plates are located at radii *ρ* = *ρ*_1_ and *ρ* = *ρ*_2_, where the inner and outer plate bear effective charges 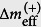 and 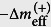, respectively. Here *ρ*_1_ (*ρ*_2_) defines the radius of the “mass-point” of positive (negative) *effective topological charge* spatial distribution.

To estimate critical conditions for which a stable pair {*defect, antidefect*} bearing unit topological charges {1/2, −1/2} is created driven by the ETCC mechanism, we calculate the total energy Δ*F* needed for this process. The critical condition is inferred from the requirement Δ*F* = 0, where penalties of forming the TDs are compensated by gains due to at least partially cancelled Δ*m*_eff_. The critical condition reads


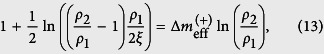


where details are given in the Supplementary Material.

### Numerical results

In this subsection we illustrate numerically the ETCC mechanism on simple demonstrative closed shapes exhibiting a spherical topology. The mechanism is particularly pronounced for cases where the *intrinsic* elastic free energy contributions are dominant, corresponding in our modelling to *k*_i_ ≫ *k*_e_. For this purpose we set *k*_e_ = 0 in the main part of our study.

We first consider the ellipsoidal shapes. Of interest to us is the impact of the spatially dependent *K* and NP-induced *virtual topological charge* on the position and number of TDs. We impose the spatially dependent *K* by varying the ratio *η* = *a*/*b* of the ellipsoids, see Eq. (7). In addition to reference configurations with Δ*m*_v_ = 0 we also analyse structures containing a different number of spatially fixed circular NPs of the radius 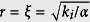, where each nanoparticle effectively acts as a topological defect bearing *m* = 1. Experimentally, such conditions could be realised by enforcing homeotropic anchoring at the NP’s interface^35^ (*i.e*. the molecules tend to be aligned along the NP’s surface normal). In Fig. S1 in the Supplementary Material, we illustrate predetermined positions of NPs used in simulations on the case of spherically shaped *ordered soft films*. These configurations possess either i) one (Fig. S1b), ii) two (Fig. S1c) or iii) three NPs (Fig. S1d). The centres of NPs are placed at i) (*u*_1_ = 0, *v*_1_ = *π*/2), ii) (*u*_1_ = 0, *v*_1_ = *π*/2), (*u*_2_ = 0, *v*_2_ = 0), iii) (*u*_1_ = 0, *v*_1_ = *π*/2), (*u*_2_ = 0, *v*_2_ = 0), (*u*_3_ = 0, *v*_3_ = *π*), where (*u*_*i*_, *v*_*i*_) locates the position of the i-th NP, see Eq. (7).

**Figure 2:**
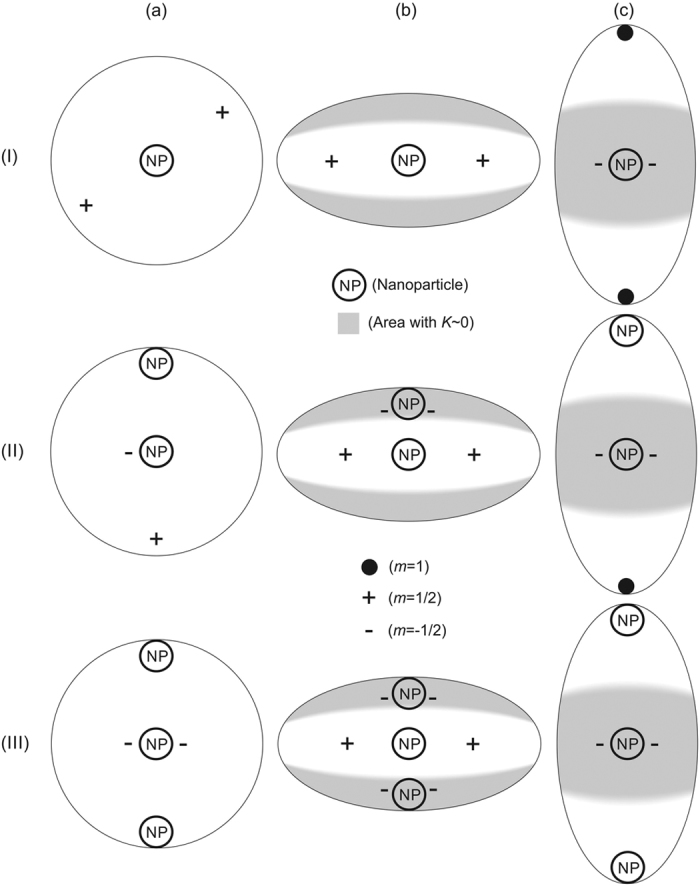
Schematic representation of ETCC limit structures on ellipsoidal shells. Approximate positions of TDs are presented on (**a**) spherical, (**b**) oblate, and (**c**) prolate ellipsoids. In cases (**I**), (**II**) and (**III**) ellipsoids host one, two, and three NPs bearing *m* = 1, respectively.

**Figure 3:**
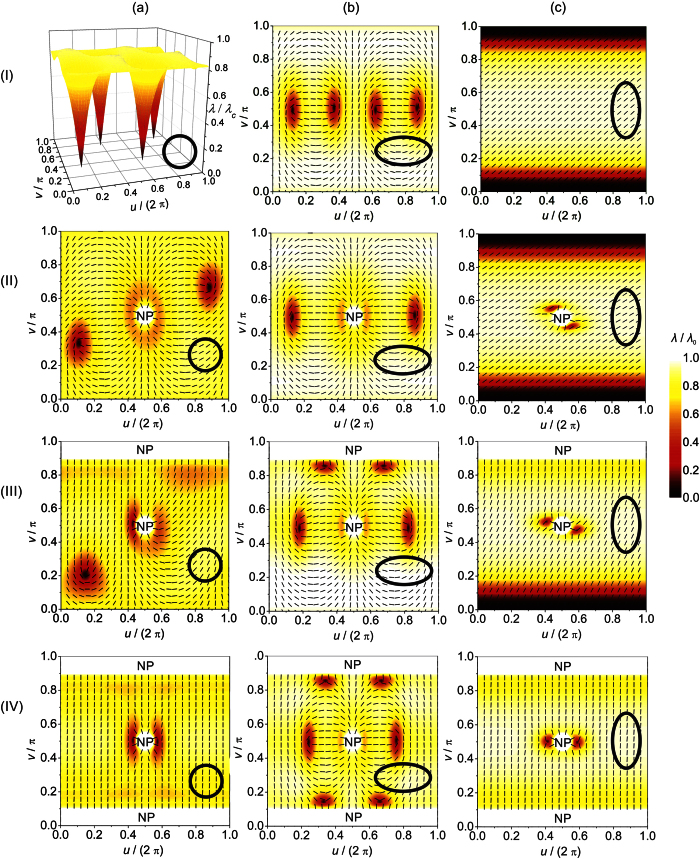
Calculated order parameter profiles in the (*u*, *v*) plane of the ellipsoidal shells. Column (**a**): spherical shapes, *a*/*b* = 1.0; Column (**b**): oblate shapes, *a* = *R* and **(I)** *b*/*a* = 1.5, **(II)** *b*/*a* = 1.5, **(III)** *b*/*a* = 2.0, **(IV)** *b*/*a* = 2.0; Column (**c**): prolate shells, *b* = *R* and **(I)** *a*/*b* = 7.0, **(II)** *a*/*b* = 5.0, **(III)** *a*/*b* = 4.5, **(IV)** *a*/*b* = 3.0. Black circles and ellipses indicate shell shapes. Nanoparticles are labelled NP. Nematic ordering was calculated for: *R*/*ξ* = 3.5, *k*_e_* *= 0, *R* = min{*a*, *b*}.

**Figure 4:**
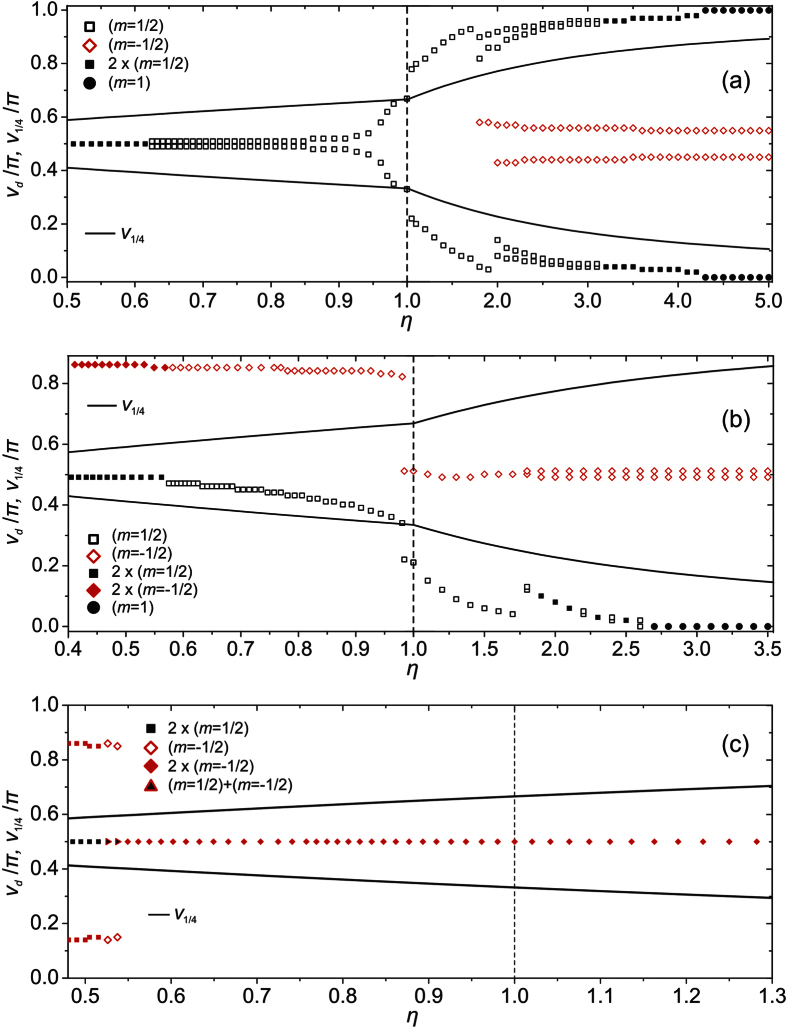
Trajectories of topological defects as the functions of *η* = *a*/*b*. By *v*_d_ we denote the *v* coordinate of the defect origin and by *v*_1/4_ the point to which the surface integral of the Gaussian curvature from the equator divided by 2*π* equals 1/4 *m*_tot_. Cases with (**a**) one, (**b**) two and (**c**) three nanoparticles are presented. *R*/*ξ *= 3.5, *k*_e_ = 0, *R* = min{*a*, *b*}.

Possible ETCC *limit structures* enforced by the electrostatic-analogy based ETCC mechanism, which is effective for *η* ≠ 1, are plotted in the (b) and (c) row of Fig. 2. The Δ*ζ*_−_ (Δ*ζ*_+_) surface patches are coloured with white (grey) color. The corresponding representative simulation results are assembled in Figs 3 and 4. In Fig. 3 we plot *λ*(*u*, *v*) dependences, from which we infer the positions of TDs. Namely, in the centre of a TD’s core, the degree of order is melted, i.e *λ* = 0. In the (a) column of Fig. 3, we plot the reference configurations calculated for spherical *ordered soft films*, while structures reached for relatively strongly oblate or prolate ellipsoids, for which ETCC *limit structures* are realised, are shown in the columns (b) and (c), respectively. In Fig. 4 we trace the zenith angular positions *v*_*d*_ of TDs on varying *η*, revealing how ETCC *limit structures* are formed.

We first describe the typical patterns of TDs in ellipsoids in the absence of NPs. Note that such configurations have already been studied in detail in^13^,^23^,^42^. Spherical *ordered soft films* exhibit in equilibrium four *m* = 1/2 as shown in Fig. 3a, line (i). On the surface of a sphere with radius *R* the Gaussian curvature is constant and equals *K* = 1/*R*^2^. Therefore, the position of TDs is dominated by their mutual repulsion. In order to maximise their separation they reside in vertices of a hypothetical inscribed tetrahedron^23^. For *η* ≠ 1 the Gaussian curvature becomes spatially dependent. In each ellipsoid, two types of different patches {Δ*ζ*_−_, Δ*ζ*_+_} emerge as discussed in the above subsection. Note that the Δ*ζ*_−_ (Δ*ζ*_+_) patch attracts TDs bearing *m* > 0 (*m* < 0). For oblate (prolate) structures Δ*ζ*_+_ patches are located at the poles (equatorial band) of structures and are characterised by 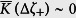. Therefore, in the limit of strong oblateness TDs are assembled roughly along the equatorial line in order to compensate for Δ*m*_K_ (Δ*ζ*_−_) < 0, see Fig. 3b, line (i). The equatorial line *K* exhibits maximal value and therefore acts as an attractor for TDs. In strongly prolate structures TDs are pushed towards the poles for the same reason. In the case shown in Fig. 3c, line (i), the characteristic Δ*ζ*_−_ region is strongly localised at the poles and consequently two *m* = 1/2 TDs merged into a single TD exhibiting *m* = 1.

Next, we treated cases possessing one NP bearing *m* = 1 as depicted in Fig. S1b. In a sphere two *m* = 1/2 TDs are present, yielding *m*_*tot*_ = 2, see Fig. 2a, line (i), and Fig. 3a, line (ii). The spatial distribution of TDs is dominated by repulsion among real and virtual topological charges, tending to maximise their mutual separation. On increasing the oblateness the two TDs progressively approach the equatorial line as shown in Fig. 4a, for which an ETCC *limit structure* is realised (see also Fig. 2b, line (i); Fig. 3b. line (ii)). By contrast, on increasing the prolateness an ETCC *limit structure* could not be realised via the existing set of *virtual* and real TDs present in the spherical geometry. In this case, two pairs {*defect, antidefect*} = {*m* = 1/2, *m* = −1/2} must be formed. The two *antidefects* are needed to screen the central virtual charge enforced by NP. On the other hand, *defects* are moved toward the poles. The corresponding ETCC *limit structure* is shown in Fig. 2c. line (i) (schematic sketch) and Fig. 3c, line (ii) (simulation). The spatial evolution of TDs and the triggering of pairs {*defect, antidefect*} on increasing *η* are shown in Fig. 4a.

If two NPs bearing *m* = 1 are present, see Fig. S1c, topological requirements do not necessitate the presence of “real” TDs. However, even in spherical geometry a pair {*defect, antidefect*} is created as demonstrated in Fig. 3a, line (iii), which can be explained roughly using an electrostatic analogy (Supplementary Material). In the oblate ETCC *limit structure* an additional pair {*defect, antidefect*} is formed, which enables the cancellation of *effective topological charges* in all surface patches as sketched in Fig. 2b, line (ii). The corresponding numerically obtained texture is shown in Fig. 3b, line (iii). One sees that the two *antidefects* move towards the upper pole to screen the NP’s *virtual topological charge*. By contrast, the *defects* assemble in the equatorial region and together with the central NP’s *virtual topological charge* compensate for the *curvature topological charge*. To form the prolate ETCC *limit structure*, a pair {*defect, antidefect*} also needs to be formed. However, in this case the *antidefects* assemble in the equatorial region to compensate for the NP’s *virtual topological charge*, see Fig. 2c, line (ii), and Fig. 3c, line (iii). On the other hand, the two *defects* assemble (and in the case studied even merge) at the bottom pole of the ellipsoid to cancel the negative *curvature charge*. The variations of the zenith positions of TDs and the creation of new pairs of TDs on varying *η* spanning an oblate and prolate ETCC *limit structure* are shown in Fig. 4b.

We proceed by studying configurations possessing 3 NPs (see Fig. S1d). To obey the Poincaré-Hopff and Gauss-Bonnet theorems, two *antidefects* are introduced in the spherical geometry. The formed structure is again understood by an electrostatic analogy. Both *antidefects* assemble close to the central NP and effectively neutralise its charge (see Fig. 2a, line (iii), and Fig. 3a, line (iv)). Therefore, in the resulting structure only the repulsive interaction between positive *virtual charges* at opposite poles remains. This configuration of TDs also persists in prolate structures (Fig. 2c, line (iii), and Fig. 3c, line (iv)), because it fulfils the ETCC mechanism tendency. By contrast, to realise oblate ETCC *limit structures* two pairs {*defect, antidefect*} need to be formed (see Fig. 2b, line (iii), and Fig. 3b, line (iv)) to compensate for the *effective topological charge* in each characteristic surface patch. Therefore, at each pole the *virtual topological charge* is compensated by two *antidefects*. On the other hand, the *curvature topological charge* of the equatorial region is compensated by two *defects* and the central NP’s *virtual topological charge*. The corresponding spatial variations of TDs on varying *η* are depicted in Fig. 4c.

**Figure 5:**
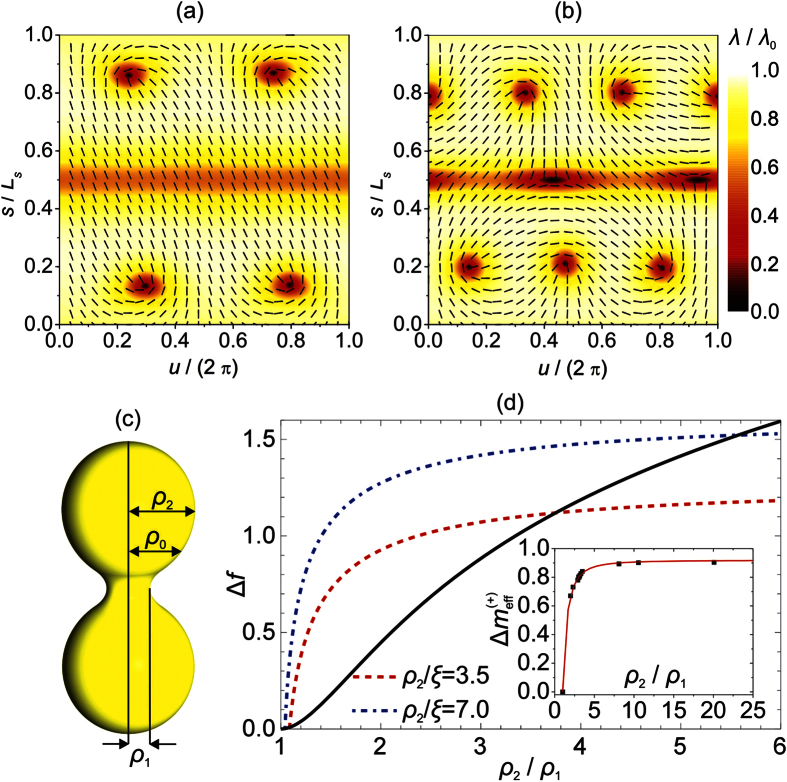
Depinning threshold in dumb-bell configurations. Panels (**a**,**b**) show nematic ordering in the (*u*, *s*) plane just below and above the threshold calculated for *ρ*_2_/*ξ* = 7, *k*_e_ = 0. Here, *s* is the arc length of the profile curve (see Eq. (14)). The shape, calculated at the threshold, is presented in panel **(c)**, where *ρ*_0_ determines the value of *ρ*(*s*) where *K* = 0. In panel **(d)** we plot the *penalty* Δ*f*^ (*p*)^ (left-hand side of Eq. (13), dashed red line (*ρ*_2_/*ξ* = 3.5), dotted dashed blue line (*ρ*_2_/*ξ* = 7)) and *gain* Δ*f *^(*g*)^ (right-hand side of Eq. (13), full line) contributions as the functions of *ρ*_2_/*ρ*_1_. In the inset we plot 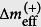 as the function of *ρ*_2_/*ρ*_1_. Squares reveal points for which 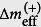 was calculated and the full line is obtained as the corresponding best fit.

We further demonstrate the curvature driven depinning of a pair {*defect, antidefect*} on the case of a dumb-bell shaped *ordered soft film* (see Fig. 5c). Different vesicle shapes were calculated within the spontaneous curvature model (i.e. we neglected the contributions of *f*_c_ and *f*_e_ in Eq. (3)) on varying the ratio *σ* = *V*/*V*_0_ and the membrane spontaneous curvature *C*_0_. Here, *V* determines the vesicle’s volume and *V*_0_ stands for the volume of the sphere exhibiting the same surface area. In the simulations, we started from a spherical vesicle, corresponding to *σ* = 1. In this geometry, the *ordered soft film* exhibits four TDs bearing *m* = 1/2 (see Fig. 3a, line (i)). On decreasing *σ*, a dumb-bell vesicle shape with an increasing ratio *μ* = *ρ*_2_/*ρ*_1_ (see Fig. 5c) is gradually formed. Note also that *C*_0_ is adequately adjusted in order to obtain shapes with inversion symmetry. The corresponding growth of 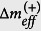
(*μ*) is depicted in the inset of Fig. 5d, where we split the upper part of a vesicle into two surface patches, characterised by *K* > 0 and *K* < 0. For a strong enough curvature in the neck area, two pairs {*defect, antidefect*} are formed due to the ETCC mechanism. In our simulations the transition occurs at the critical value *μ*_c_ = 3.06 ± 0.01, where order parameter profiles in the (*u*, *s*) plane just below and above the threshold are plotted in Fig. 5a,b, respectively. Here, *s* is the arc length of the profile curve characterising axial-symmetric shapes (see Methods). The shape shown in Fig. 5c is calculated at the depinning threshold. In Fig. 5d we analyse our estimate derived based on the electrostatic analogy (see Eq. (13)). We plot the *penalty* (
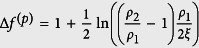
, dashed red line (*ρ*_2_/*ξ* = 3.5), dotted dashed blue line (*ρ*_2_/*ξ* = 7)) and *gain* (
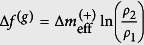
, full line) contributions on increasing *ρ*_2_/*ρ*_1_. The crossing of the lines corresponds to the critical condition. In this estimate we set that *ρ*_2_ (*ρ*_1_) is equal to the maximal (minimal) radius of the structure in the azimuthal plane. Using the calculated 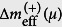, the estimate (Eq. (13)) yields *μ*_*c*_ = 4 ± 1.5 which is in rough agreement with the numerically obtained result.

## Conclusions

We studied theoretically and numerically the impact of the Gaussian curvature on the position and number of topological defects in 2D nematic orientational ordering. We introduced the *effective topological charge* Δ*m*_eff_ in surface patches characterised by a distinctive spatially averaged Gaussian curvature 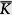. We demonstrated the tendency Δ*m*_eff_ (Δ*ζ*) → 0 in each patch, to which we refer as the ETCC *limit structure*. The effectiveness of the *Effective Topological Charge Cancellation* (ETCC) mechanism increases with the increasing value of |Δ*m*_eff_| and becomes apparent when |Δ*m*_eff_| becomes comparable with the unit topological charge *m*_0_ of the system (in our case *m*_0_ = ±1/2). The mechanism is most evident for systems exhibiting weak *extrinsic* elasticity. The impact of the latter is demonstrated in Figure S3 in the Supplementary Material.

The ETCC mechanism could be exploited for a controlled positional targeting of TDs to predetermined locations. This property could be exploited in various photonic materials. For example, by changing a local curvature, a local property of such a medium could be dramatically changed by dragging TDs in or out, which could be exploited for local information manipulation. Furthermore, TDs in general efficiently trap various NPs owing to the Defect Core Replacement mechanism^4^,^37^. That is to say, if NPs do not strongly locally disrupt the gauge (in our study*the nematic director*) field, which exhibits TDs, then NPs tend to assemble within the cores of TDs. In such a way the condensation penalty of a relatively expensive core is reduced. Therefore, via TDs, one could positionally control trapped NPs, which could act as carriers of additional material or a functional property of the system. The manipulation of TDs and NPs could also be exploited for various self-assembling strategies. For example, as suggested by Nelson^11^, objects studied in our work might be exploited as meta-atoms with a self-assembling ability to form crystal-like structures within isotropic fluids containing nano-binders. In this case, TDs determine the “valence” of meta-atoms because of their potential ability to efficiently locally pin nano-binders. Via them nearby meta-atoms could be physically bonded. Controlling the positions and the number of TDs in such shells is expected to enable a self-assembling of meta-atoms into crystal configurations with almost arbitrary symmetries. Note that symmetry often imposes a dominant impact on the effective physical properties of materials.

## Methods

In simulations, we calculated the nematic ordering for a chosen geometry of a closed two-dimensional surface. For demonstrative purposes we considered axial-symmetric shells with inversion symmetry. We assumed that the elastic costs of bending shells are large with respect to elastic costs related to deformations in the orientational degree of order. We considered either ellipsoidal shells using Eq. (7) or dumb-bell shaped shells calculated using the spontaneous curvature model. We first summarised the main ingredients of the spontaneous curvature model. Afterwards, we described the 2D Landau-de Gennes approach by which the orientational ordering was calculated for a given shell geometry.

### Calculation of shapes

In generating shell shapes calculated within the spontaneous curvature model, we introduce arc length *s* of the profile curve and angle *ϕ* (*s*), which represents the angle of the tangent to the profile curve with the plane that is perpendicular to the axis of rotation **e**_z_. The shell profile curve is calculated by^48^:


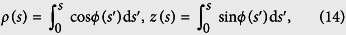


where *ρ*(*s*) and *z* (*s*) are the coordinates of the profile in the (*ρ*, *z*)-plane. The surface of the shell is constructed by the rotation of the profile curve about the **e**_*z*_ axis by an angle of *u* = 2*π*. For closed and smooth surfaces, we have to apply the following boundary conditions: *ϕ*(0) = 0, *ϕ*(*L*_s_) = *π* and *ρ*(0) = *ρ*(*L*_s_) = 0, where *L*_s_ is the profile length. The function describing the angle *ϕ*(*s*) is approximated by the Fourier series^48^,


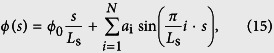


where *N* is the number of Fourier modes, *a*_i_ are the Fourier amplitudes, and *ϕ*_0_ is the angle at the north pole of the shell, *ϕ*_0_ = *ϕ*(*L*_s_) = *π*. The local principal curvatures *C*_1_ and *C*_2_ are given as 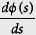 and 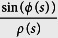, respectively. The bending energy density *f*_b_ (see Eq. (4)) is therefore a function of the Fourier amplitudes *a*_i_ and the shape profile length *L*_s_. Equilibrium vesicle shapes are calculated by the numerical minimisation of the function of many variables^48^. In the minimisation procedure, the vesicle surface area and the volume are kept constant.

### Calculation of nematic order

We parameterise the nematic order tensor as^42^,^43^:


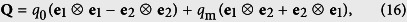


where *q*_0_ and *q*_m_ are scalar functions. We assume that shell surface *ζ* is a surface of revolution with rotational symmetry about the *z*-axis within the Cartesian system (*x*, *y*, *z*). We represent the position vector **r** of a generic point lying on *ζ* as:


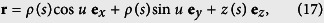


where *u* ∈ [0, 2*π*] stands for the azimuthal angle. Eq. (17) represents a general description of an axi-symmetric surface. In our study, we focused on two types of axi-symmetric surfaces. Ellipsoidal surfaces are parameterised by Eq. (7), while the surfaces defined by the profile curve are described by Eq. (14). The following mathematical description of various surface properties is valid for both types of surfaces, although it is adjusted to the surfaces defined by the profile curve.

The standard functions that represent the first fundamental form on surface *ζ* in the (*u*, *s*) coordinates are given by^42^,^49^:


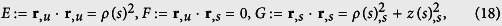


where a comma denotes the differentiation and


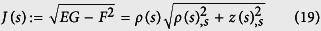


is the Jacobian determinant. On a surface of revolution parallels and meridians are lines of principal curvature. We define **e**_1_ as a unit vector along meridians (*u* = const) and **e**_2_ as a unit vector along parallels (*s* = const). The meridians are also geodesics, so their geodesic curvature *κ*_g1_ = 0. The geodesic curvature of the parallels can be written as^42^,^49^:


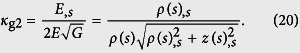


The Gaussian and mean curvatures of the shell surface *ζ* are^42^,^49^:


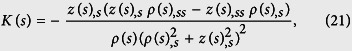



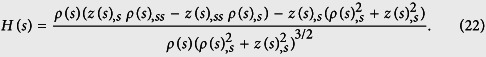


*K* and *H* are connected to the local principal curvatures *C*_1_ and *C*_2_ via


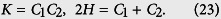


The surface gradient of scalar function *ψ* in the coordinates (*u*, *s*) on surface *ζ* is given by:


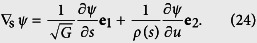


The surface gradients of **e**_1_ and **e**_2_ are given by Eqs (S4) and (S5) (see Supplementary Material), respectively. We express the free energy density in terms of fields *q*_0_ and *q*_m_ for a given *ordered soft film* geometry. The equilibrium textures were calculated either by using the standard Monte Carlo method or by numerically solving the Euler-Lagrange equations^42^,^49^. Both methods yield the same results.

## Additional Information

**How to cite this article**: Mesarec, L. *et al*. Effective Topological Charge Cancelation Mechanism. *Sci. Rep*. **6**, 27117; doi: 10.1038/srep27117 (2016).

## Supplementary Material

Supplementary Information
