# Supplementary material for: Effective Topological Charge Cancelation Mechanism
Source: Sci Rep. 2016 Jun 1;6:27117. doi: 10.1038/srep27117 (PMC4890433; doi:10.1038/srep27117)
Supplement: Supplementary Information [file srep27117-s1.pdf]

Effective Topological Charge Cancelation Mechanism

Luka Mesarec,[1,*] Wojciech Góźdź,[2] Aleš Iglič,[1] and Samo Kralj[3,4,5,†]

[1] *Laboratory of Biophysics, Faculty of Electrical Engineering, University of Ljubljana, Tržaška 25, SI-1000 Ljubljana, Slovenia*
[2] *Institute of Physical Chemistry, Polish Academy of Sciences, Kasprzaka 44/52, 01-224 Warsaw, Poland*
[3] *Condensed Matter Physics Department, Jožef Stefan Institute, Jamova 39, 1000 Ljubljana, Slovenia*
[4] *Jožef Stefan International Postgraduate School, Jamova 39, 1000 Ljubljana, Slovenia*
[5] *Faculty of Natural Sciences and Mathematics, University of Maribor, Koroška 160, 2000 Maribor, Slovenia*

PACS numbers:

Supplementary Material

In-plane ordering

Note that our approach could be applied also to cases possessing out-of-plane orientational ordering. For example, for LC molecules, represented by the unit director field **n,** which are tilted with respect to the local surface normal **v**, the in-plane ordering is determined by a vector order parameter field [1] $\mathbf{m} = \mathbf{n} - (\mathbf{v} \cdot \mathbf{n})\mathbf{v}$. In this case, **m** is the relevant vector field addressed by the Gauss-Bonnet and Poincaré-Hopff theorem.

Furthermore, there are numerous examples in which in-plane orientational ordering exists in biological membranes. Below, we summarise a few examples.

In-plane membrane ordering in the membranes due to the lipid tilt, chirality and other mechanisms was considered first by Helfrich, Lubensky and Prost in the late nineteen-eighties [2, 3].

The in-plane ordering in the curved biological or lipid membranes is coupled to local membrane curvature and driven by the anisotropic shape of membrane components (proteins or lipids). The in-plane orientational ordering of anisotropic membrane proteins favours anisotropic membrane curvature, i.e. non-zero local curvature deviator (see for example [4–9]. Coupling between the in-plane orientational ordering and the membrane local curvature may lead to the formation of highly curved anisotropic membrane regions like nanotubular membrane protrusions or membrane necks, connecting a parent cell to a daughter vesicle [4, 9–12]. A possible physiological role of topological defect in the highly curved regions of membrane neck with high in-plane ordering has recently been discussed by Jesenek et al. [13, 14].

Anisotropic membrane proteins can be embedded in the membrane or attached to the membrane surface. Typical anisotropic proteins (attached to the membrane surface) are banana-like shaped BAR-domain proteins which may, due to their in-plane ordering [15–17], stabilise thin membrane protrusions [18].

The anisotropic shape of lipid molecules may lead to their in-plane (nematic) ordering in highly curved membrane regions. Recently, it was pointed out [19] that the concept of the anisotropic shape of lipid molecules and their in-plane ordering may better explain the L_α - inverted hexagonal HII phase transition and the stability of the HII-phase at higher temperatures than the concept of isotropic lipid shapes. A similar idea was also expressed earlier [20], but was not applied to any model calculations. The in-plane orientational ordering in (hexatic) membranes has been theoretically considered also by Nelson and Peliti [21], David et al. [22], and Park and Lubensky [23].

Fournier and Galatola [24] studied theoretically the effect of surfactant polar head on the membrane in-plane ordering. In accordance, experiments and theoretical considerations [10] showed that anisotropic dimeric detergents or detergents with dimeric headgroup induce the growth of highly curved tubular membrane protrusions (and the release of tubular daughter vesicles), which is not possible with isotropic detergents that induce only spherical membrane protrusions (and release spherical daughter vesicles).

---

[*] Electronic address: `mesarec.luka@gmail.com`
[†] Electronic address: `samo.kralj@um.si`

Intrinsic and extrinsic elastic contributions

Below, we present the minimal model with which we demonstrate the key difference between the *intrinsic* and the *extrinsic* elastic contributions in describing orientational ordering on curved surfaces. Afterwards, we illustrate the typical impact of the *extrinsic* curvature contribution on nematic structures within nematic ellipsoidal shells, considering the tensor order parameter $\mathbf{Q}$ (Eq. (2)) and the free energy contributions introduced in Eq. (5) and Eq. (6).

Minimal model

In general, an elastic free energy penalty of an elastic medium living within a 2D curved surface consists of the so-called *intrinsic* and *extrinsic* contributions [25–27]. To illustrate their role, we consider the simplest possible elastic free energy term

$$f_{\mathrm{e}} = k\left|\nabla_{\mathrm{s}}\mathbf{n}\right|^2. \tag{S1}$$

Here, $\mathbf{n}$ is a unit vector field lying within a surface, k is a positive elastic constant, and ∇_{s} stands for the surface gradient [28]. The latter is related to the conventional 3D gradient operator via $\nabla_{\mathrm{s}} = (\mathbf{I} - \mathbf{v}\otimes\mathbf{v})\nabla$. We express $\mathbf{n}$ in the surface principal curvature direction frame $\{\mathbf{e}_1,\mathbf{e}_2\}$ as

$$\mathbf{n} = \mathbf{e}_1\cos\theta + \mathbf{e}_2\sin\theta. \tag{S2}$$

where θ is the angle between the unit vector field $\mathbf{n}$ and the first principal curvature direction $\mathbf{e}_1$. The local surface curvature C, seen by the molecule aligned along $\mathbf{n}$, can be expressed by the Euler relation as: $C = C_1\cos^2\theta + C_2\sin^2\theta$. It follows

$$\nabla_{\mathrm{s}}\mathbf{n} = \cos\theta\nabla_{\mathrm{s}}\mathbf{e}_1 - \sin\theta\mathbf{e}_1\otimes\nabla_{\mathrm{s}}\theta + \sin\theta\nabla_{\mathrm{s}}\mathbf{e}_2 + \cos\theta\mathbf{e}_2\otimes\nabla_{\mathrm{s}}\theta. \tag{S3}$$

Taking into account [28]

$$\nabla_{\mathrm{s}}\mathbf{e}_1 = \kappa_{\mathrm{q}1}\mathbf{e}_2\otimes\mathbf{e}_1 + \kappa_{\mathrm{q}2}\mathbf{e}_2\otimes\mathbf{e}_2 - C_1\mathbf{v}\otimes\mathbf{e}_1, \tag{S4}$$

$$\nabla_{\mathrm{s}}\mathbf{e}_2 = -\kappa_{\mathrm{q}1}\mathbf{e}_1\otimes\mathbf{e}_1 - \kappa_{\mathrm{q}2}\mathbf{e}_1\otimes\mathbf{e}_2 - C_2\mathbf{v}\otimes\mathbf{e}_2, \tag{S5}$$

where $\kappa_{\mathrm{q}1}$ ($\kappa_{\mathrm{q}2}$) are geodesic curvatures along $\mathbf{e}_1$ ($\mathbf{e}_2$), we obtain

$$\left|\nabla_{\mathrm{s}}\mathbf{n}\right|^2 = \left|\nabla_{\mathrm{s}}\theta + \mathbf{A}\right|^2 + \mathbf{n}\cdot\mathbf{C}^2\mathbf{n}. \tag{S6}$$

The quantity

$$\mathbf{A} = \kappa_{\mathrm{q}1}\mathbf{e}_1 + \kappa_{\mathrm{q}2}\mathbf{e}_2 \tag{S7}$$

is the so called spin connection and it holds [26, 29] $K = |\nabla\times\mathbf{A}|$.

The first contribution in Eq. (S6) is referred to as the *intrinsic* term. It enforces the spatially non-homogeneous orientation of $\mathbf{n}$ if $K \neq 0$, which introduces a geometric frustration into the system. It derives from the incompatibility of parallel and straight directions on surfaces with Gaussian curvature. Note that using the covariant derivative approach in expressing elastic free energy contributions yields only *intrinsic*-type contributions. The covariant derivative is defined via parallel transport. While parallel transported $\mathbf{n}$ experiences minimal distortions, it holds [28]

$$\nabla_{\mathrm{s}}\mathbf{n} = -\left(\mathbf{v}\otimes\nabla_{\mathrm{s}}\mathbf{v}\right)\mathbf{n}. \tag{S8}$$

If $\mathbf{n}$ is parallel transported along a closed path, then the difference in orientation of $\mathbf{n}$ before and after such transport reveals a geometric frustration. Taking into account Eq. (S3) on the left side of Eq. (S8) and considering $\nabla_{\mathrm{s}}\mathbf{v} = C_1\mathbf{e}_1\otimes\mathbf{e}_1 + C_2\mathbf{e}_2\otimes\mathbf{e}_2$ one obtains $\nabla_s\theta = -\mathbf{A}$. Therefore, the *intrinsic* term in Eq. (S6) is minimised if $\mathbf{n}$ is parallel transported. Such a configuration represents a local ground state, exhibiting minimal possible elastic free energy distortions.

The second term in Eq. (S6) is referred to as the *extrinsic* contribution. Its influence is reminiscent of an external orientational field enforcing its orientation to $\mathbf{n}$. Expressing the term in the principal curvature frame yields

$$\mathbf{n}\cdot\mathbf{C}^2\mathbf{n} = C_1^2\cos^2\theta + C_2^2\sin^2\theta. \tag{S9}$$

One sees that for $k > 0$ this term is minimised if $\mathbf{n}$ is aligned along the principal direction exhibiting lower curvature.

The energy term (Eq. (S9)) is closely related to the energy term f_H, commonly used to study biological membranes [30], thin plates [31, 32] with anisotropic properties, and the orientational ordering of anisotropic components in biological membranes [7, 11, 33]

$$f_H = \frac{K_1}{2}(\mathrm{Tr}\mathbf{M})^2 + K_2 \mathrm{Det}\mathbf{M}, \tag{S10}$$

where K_1 and K_2 are (positive) constants. The mismatch tensor is defined as $\mathbf{M} = \mathbf{R}\mathbf{C}_m\mathbf{R}^{-1} - \mathbf{C}$, where the tensor $\mathbf{C}_m$ describes the *intrinsic* curvature of the inclusion. The rotational matrix $\mathbf{R}$ describes the rotation of the system by angle θ, which is the angle of rotation of the membrane element relative to the first principal direction $\mathbf{e}_1$. In the case of non-curved rod-like molecule the energy term (Eq. (S10)) is minimised if the molecule is aligned along the principal direction exhibiting lower curvature, which is also true for the energy term in Eq. (S9).

Therefore, the *intrinsic* elastic component is associated with variations of $\mathbf{n}$ living in 2D curved space. On the other hand, the *extrinsic* elastic component tells how $\mathbf{n}$ is embedded in 3D space. The difference between these contributions is well visible in an infinitely long cylinder of radius R. The *intrinsic* elastic penalty equals to zero for $\mathbf{n}$, pointing either along the symmetry axis or at right angles with it. To show this, we use parametrisation defined in Eq. (S2), where $\mathbf{e}_1$ is aligned along the symmetry axis. It holds $\kappa_{q1}=\kappa_{q2}= \mathbf{A} = \nabla_s\theta = 0$ for both orientations, and consequently the *intrinsic* term equals zero (see Eq. (S6)). On the contrary, the *extrinsic* term is different for these orientations due to different values of principal curvatures; namely, $C_1 = 0$ and $C_2 = 1/R$. Consequently, it forces $\mathbf{n}$ to align along $\mathbf{e}_1$.

Impact of extrinsic term

In the core of the paper, we calculated TDs in oblate and prolate shells in the presence of different number of NPs in the absence of extrinsic term. The positions of NPs are depicted in Figure S1 on cases of spherical shells, where we superimpose $\mathbf{n}$ and spatial variations in λ.

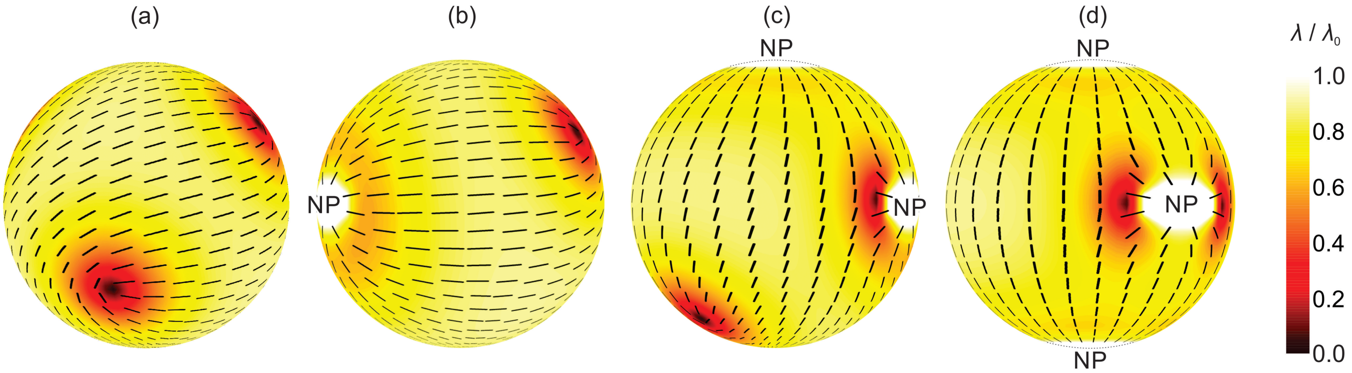

Figure S1. Nematic ordering on spherical shells. The panels **(a)**, **(b)**, **(c)** and **(d)** show cases with no, one, two and three nanoparticles, which are denoted by "NP". Each NP effectively acts as a topological defect, bearing $m = 1$. Superimposed are λ/λ_0 and the vector field $\mathbf{n}$ spatial variations. Configurations were calculated for $a/b = 1$, $a/\xi = 3.5$, $k_e = 0$.

The impact of the orientation of $\mathbf{n}$ on the extrinsic free energy contribution is depicted in Figures S2. In Figure S2a, we plot $g_e = f_e/(k_e\lambda_0^2)$ as the function of zenith angle v for prolate and oblate shells for either $\mathbf{n}$ along $\mathbf{e}_1$ (meridians, full lines) or $\mathbf{n}$ along $\mathbf{e}_2$ (parallels, thin lines). The plots which show extreme cases and variation for the arbitrary orientation of $\mathbf{n}$ are presented in Figure S2b (a prolate shell) and Figure S2c (an oblate shell). The *extrinsic* contribution effectively acts as an external field which for $k_e > 0$ favours the orientation of $\mathbf{n}$ along lines, exhibiting minimal curvature. One sees that in both geometries the *extrinsic* field is absent at $v = 0$ and $v = \pi$, where $C_1 = C_2$. In between, the field is different from zero. In prolate shells, it prefers an alignment along meridians and is in general significant for all values of $v \in [0, \pi]$ for a large enough value of k_e. On the contrary, in oblate geometries it enforces the alignment along parallels. In this case, the *extrinsic* field tends to be localised at the equatorial region, which progressively narrows on increasing the ratio b/a. Of our interest is the impact of a relatively strong *extrinsic* field (e.g., $k_e/k_i = 1$) on the number and position of TDs in ETCC *limit structures.* In prolate shells, structures are

qualitatively similar, because the *extrinsic* field does not introduce any additional frustrations. However, in oblate structures the *extrinsic* field can strongly modify patterns, because it tends to expel TDs from the equatorial region. This is depicted in Figures S3. The corresponding textures for $k_e = 0$ are plotted in the column (b) of Figure 3.

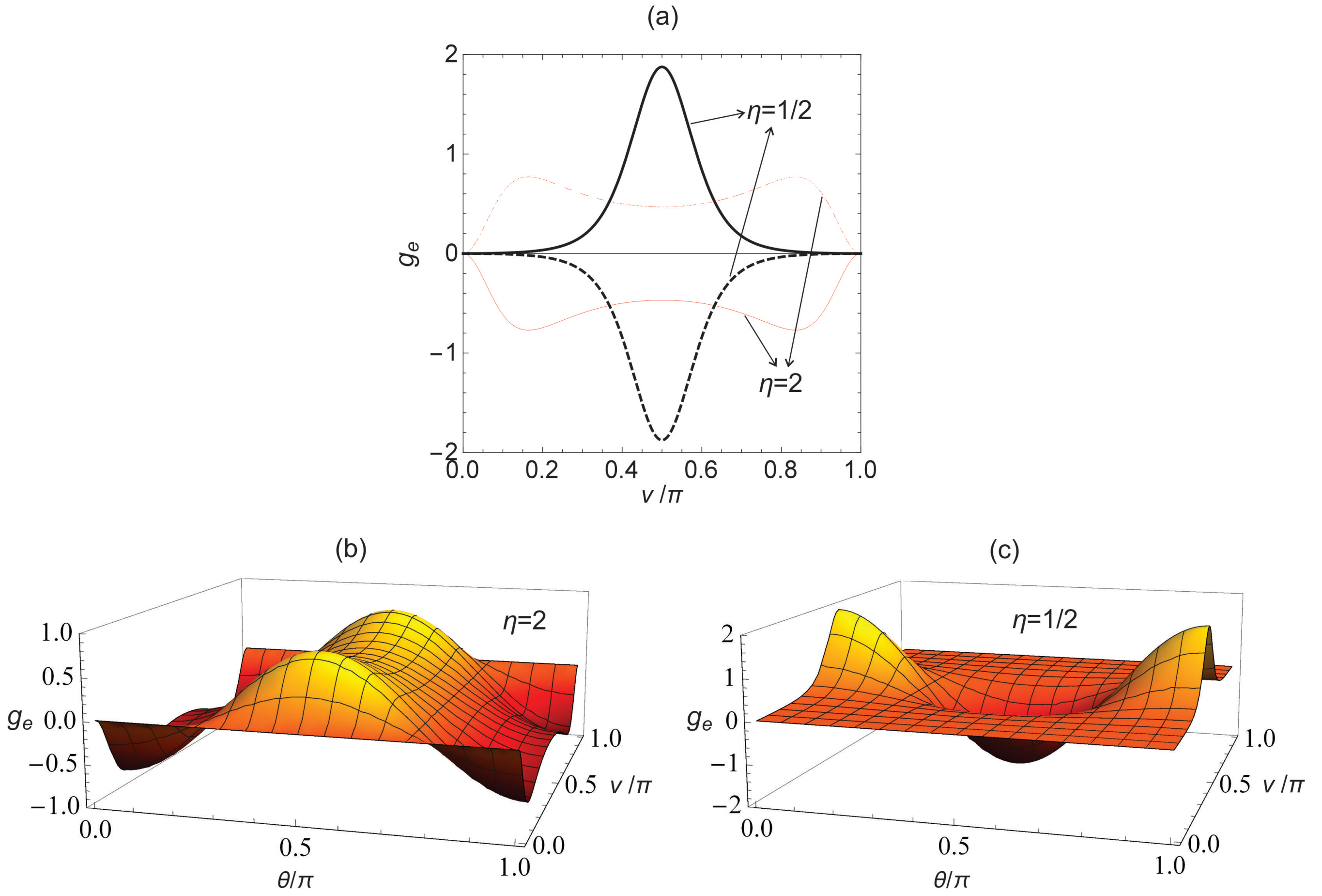

Figure S2. Spatial variations of extrinsic free energy term contribution on a prolate and oblate shell. **(a)** The extrinsic contribution to the free energy $g_e = f_e/(k_e\lambda_0^2)$ as the function of v, where thin lines represent a prolate shell ($a/b = 2$) and thick lines an oblate shell ($a/b = 1/2$). Full lines correspond to the alignment of $\mathbf{n}$ along $\mathbf{e}_1$ (meridians) while dashed lines correspond to the alignment of $\mathbf{n}$ along $\mathbf{e}_2$ (parallels). Plots $g_e(v, \theta)$ for **(b)** $a/b = 2$ and **(c)** $a/b = 1/2$. Here, θ describes the angle between $\mathbf{n}$ and $\mathbf{e}_1$.

Electrostatic analogy

Here, we sketch key steps in developing electrostatic analogy which enables us to estimate the threshold condition to form pairs $\{$*defect,antidefects*$\}$. Furthermore, we schematically visualise the depinning process for two qualitatively different geometries.

Critical condition

We first consider a flat liquid crystalline film in the Cartesian coordinates (x, y), and the free energy, which is given by Eq. (6). We neglect spatial variations in λ, set $k_e = 0$, and express the director field using Eq. (S2), where unit vectors $\mathbf{e}_i$ point along the Cartesian coordinates. If follows $f = f_0 + k\lambda_0^2 |\nabla_s\theta|^2$ where f_0 is a constant. The corresponding Euler Lagrange equation reads $\nabla_s^2\theta = 0$. Singular solutions, corresponding to topological defects

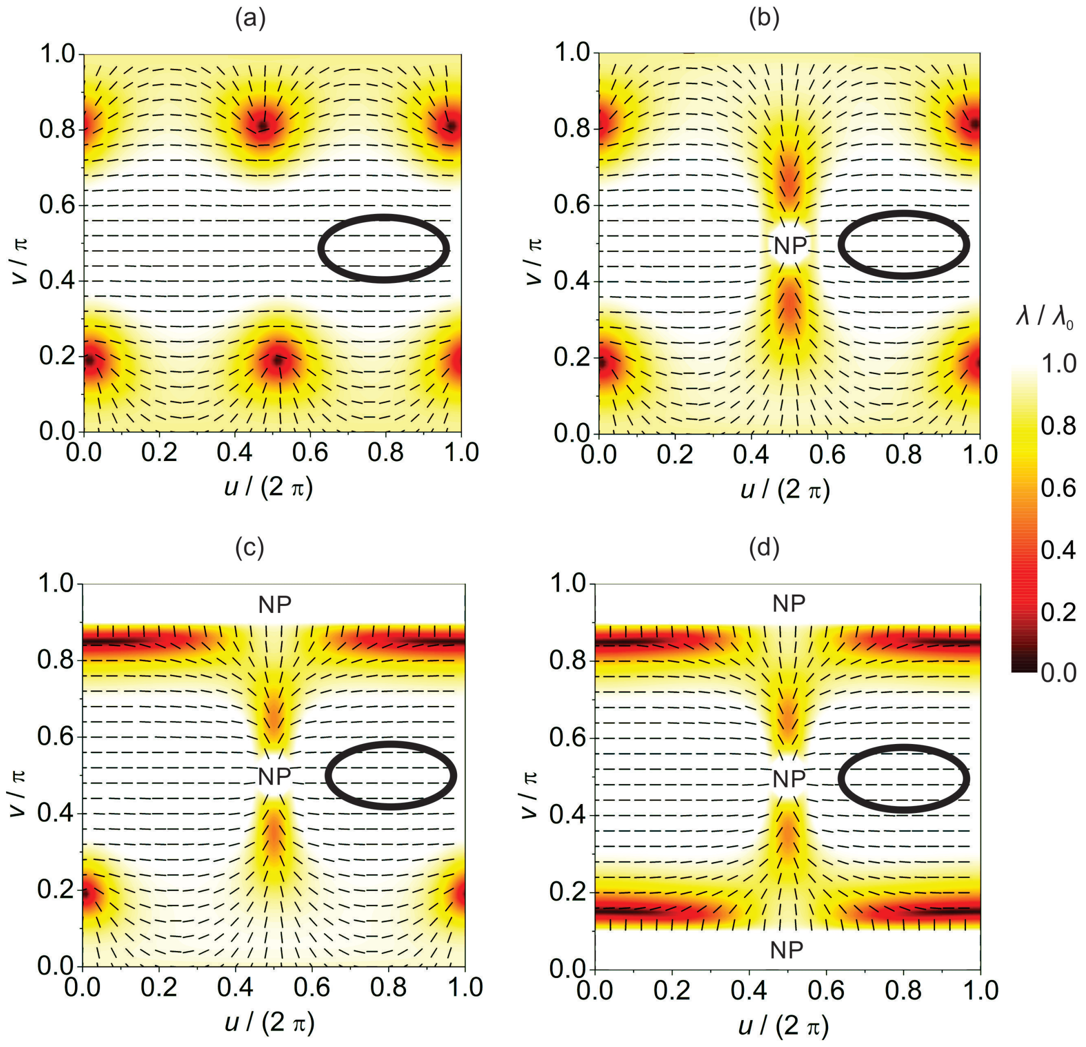

Figure S3. Impact of extrinsic term on order parameter profiles is the (u, v) plane on oblate ellipsoidal shells. (a) $b/a = 1.5$, **(b)** $b/a = 1.5$, **(c)** $b/a = 2.0$, **(d)** $b/a = 2.0$. Black ellipses indicate shell shapes. Nanoparticles are labelled by NP. Nematic ordering was calculated for: $a/\xi = 3.5$, $k = k_e$.

localised at (x_i, y_i), can be expressed as

$$\theta_i = m_i ArcTan\left(\frac{y - y_i}{x - x_i}\right) + c. \tag{S11}$$

Here, c is a constant and m_i is the winding number. We consider a pair consisting of $\{$*defect,antidefect*$\}$=$\{m_1 = m, m_2 = -m\}$ placed at $(x_1 = -\rho/2,\ y_1 = 0)$ and $(x_2 = \rho/2,\ y_2 = 0)$, respectively. The orientational pattern of the resulting structure is determined by $\theta = \theta_1 + \theta_1$. The spatial integral of the corresponding free energy yields the interaction potential

$$w_{\text{int}} = 2\pi m^2 k_F \ln(\rho/\rho_c) \tag{S12}$$

between TDs separated for a distance ρ. Here $k_F = k\lambda_0^2$ and $\rho_c \sim \xi$ is the cut-off radius estimating a typical defect's core size. In our model notations it roughly holds

$$k_{\text{F}}/(\alpha\lambda_0^2) \sim \xi^2. \tag{S13}$$

The magnitude of the corresponding attractive force per unit length is then $f_{\text{int}} = 2\pi m^2 k_{\text{F}}/\rho$. We define the *elastic electric field* via $f_{\text{int}} \equiv m E_{\text{e}}$. Therefore, a topological defect bearing topological charge m creates an elastic field of

strength

$$E_e = \frac{2\pi k_{\mathrm{F}} m}{\rho}. \tag{S14}$$

Next, we estimate energies needed to form a pair $\{m = \frac{1}{2}, m = -\frac{1}{2}\}$ of TDs at line $K = 0$ which then "fall" on relevant *capacitor* plates. We express the total free energy cost for this process as

$$\Delta F = \Delta F_{\mathrm{cond}} + \Delta F_{\mathrm{work}} + \Delta F_{\mathrm{gain}}. \tag{S15}$$

Here, ΔF_{cond} describes the free energy costs to form a pair $\{1/2, -1/2\}$ of TDs, ΔF_{work} corresponds to the work needed to separate the newly born pair, and ΔF_{gain} describes the free energy gain due to the "fall" of TDs within the capacitor's *elastic electric field.*

We suppose that $E(\Delta m_{\mathrm{eff}})$ is strong enough to trigger off TDs at an arbitrary point along the line where $K = 0$. The corresponding penalty is roughly given by the condensation free energy cost

$$\Delta F_{\mathrm{cond}} \approx \alpha \lambda_0^2 \pi \xi^2. \tag{S16}$$

The work needed to pull apart the new-born defects with charges $m = \pm\frac{1}{2}$ from the initial separation ξ to the final separation $\rho_2 - \rho_1$ overcoming their mutual attraction is equal to

$$\Delta F_{\mathrm{work}} \approx \int_{2\xi}^{\rho_{2t}-\rho_1} |m|\, E_{\mathrm{e}}(m) d\rho = \frac{1}{2}\pi k_{\mathrm{F}} \ln\left(\frac{\rho_2 - \rho_1}{2\xi}\right). \tag{S17}$$

Finally, the energetic gain is estimated by

$$\Delta F_{\mathrm{gain}} \approx -\int_{\rho_1}^{\rho_2} |m|\, E_e(\Delta m_{\mathrm{eff}}) d\rho = -\pi k_{\mathrm{F}} \Delta m_{\mathrm{eff}}^{(+)} \ln\left(\frac{\rho_2}{\rho_1}\right). \tag{S18}$$

The critical condition to form a stable pair is estimated by $\Delta F = 0$, yielding Eq. (13).

Depinning event

We next schematically illustrate events above and below the critical depinning threshold for two qualitatively different geometries, emphasising the role of the *effective topological charge.* We consider (i) dumb-bell and (ii) sphero-cylindrical shells. In the latter case we also introduce a NP acting as a "virtual" TD bearing a *virtual topological charge* $\Delta m_{\mathrm{v}} = 1$. Examples of a "real" and "virtual" charge are schematically depicted in Figure S4a and Figure S4b, respectively.

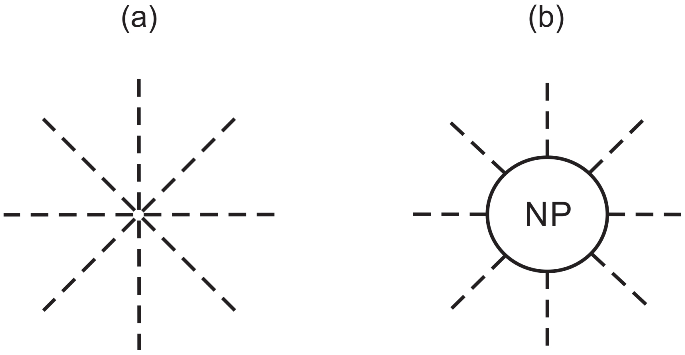

Figure S4. Schematic presentation of (a) "real" and (b) "virtual" topological defect, bearing $m = 1$.

We first consider configurational changes in geometry, depicted in Fig. 1c. Let us assume that the structure is mirror symmetric (the up and down parts of the dumb-bell are the same) and that it possesses four $m = 1/2$

defects as predicted by Poincaré-Hopff and Gauss Bonnet theorem. For a better visualisation of estimates let us assume that the dumb-bell structure consists of nearly two spherical parts connected by a narrow neck. Note that for a closed sphere surface ζ it holds $\Delta m_{\mathrm{K}}(\zeta) = -\frac{1}{2\pi}\int\int_{\zeta} K d^2\mathbf{r} = -2$. We focus, henceforth, to the upper part of the dumb-bell and consider patches $\Delta\zeta_+$ and $\Delta\zeta_-$ defined in Fig. 1c. Below the depinning threshold the two $m = 1/2$ are assembled in the region exhibiting positive Gaussian curvature because this region effectively acts as a smeared negative *curvature topological charge.* The resulting configuration is depicted in Figure S5a. The effective topological charge in the upper $\Delta\zeta_-$ patch equals to $\Delta m_{\mathrm{eff}}(\Delta\zeta_-) = \Delta m + \Delta m_{\mathrm{v}} + \Delta m_{\mathrm{K}} \sim 1+0-2 = -1$, where the used approximation is $\Delta m_{\mathrm{K}}(\Delta\zeta_-) \sim -2$. Furthermore, according to the Poincaré-Hopff and Gauss Bonnet theorem it holds $\Delta m_{\mathrm{K}}(\Delta\zeta_-) + \Delta m_{\mathrm{K}}(\Delta\zeta_+) = -1$, consequently $\Delta m_{\mathrm{K}}(\Delta\zeta_+) \sim 1$ and $\Delta m_{\mathrm{eff}}(\Delta\zeta_+) = \Delta m + \Delta m_{\mathrm{v}} + \Delta m_{\mathrm{K}} \sim 0+0+1 = 1$. The upper dumb-bell part is therefore roughly equivalent to the capacitor shown in Figure 1d, where the plates at $\rho = \rho_1$ and $\rho = \rho_2$ bear charges $\Delta m_{\mathrm{eff}}(\Delta\zeta_+) \sim 1$ and $\Delta m_{\mathrm{eff}}(\Delta\zeta_-) \sim -1$, respectively. If two pairs $\{$*defect,antidefect*$\}$ are created, then the capacitor plates are partially discharged, as shown in Figure S5b. The complete discharging of the capacitor, corresponding to the ETCC limit structure, requires a formation of four pairs $\{$*defect,antidefect*$\}$.

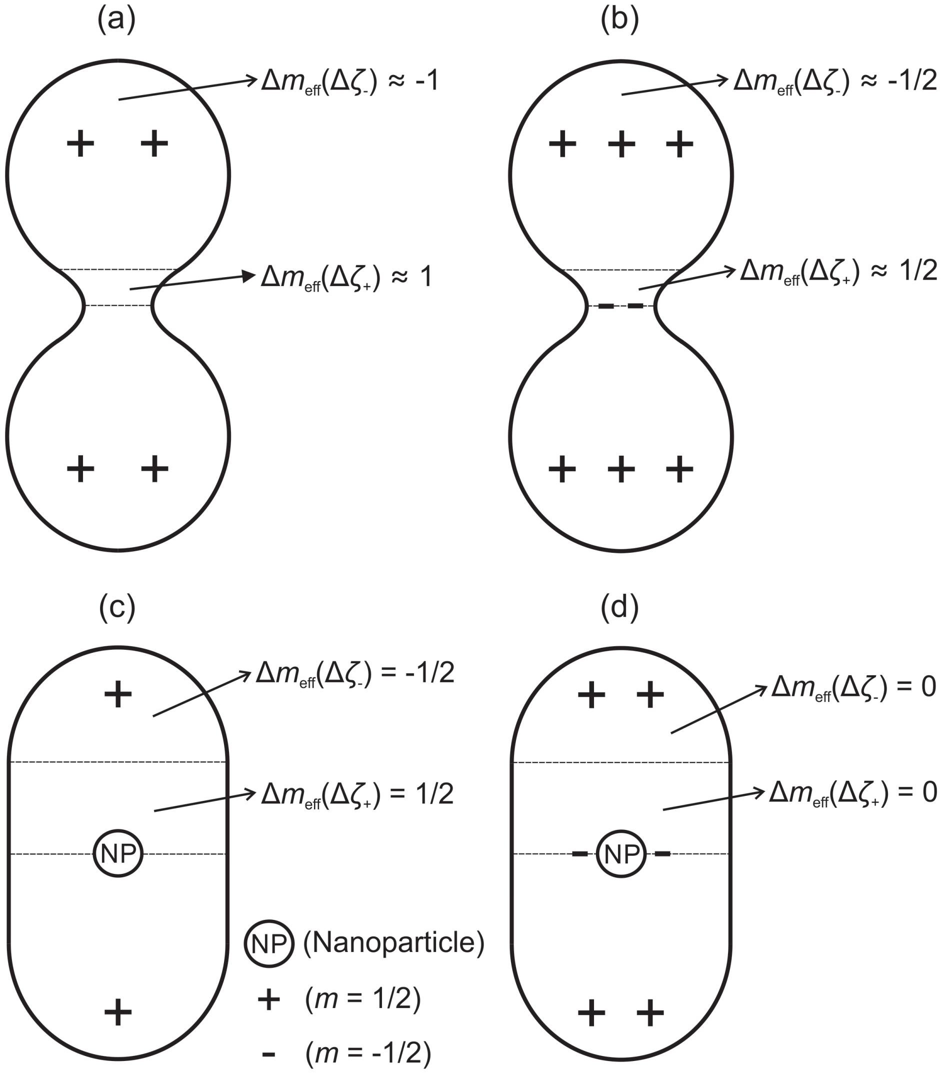

Figure S5. Schematic illustration of equilibrium configurations above (b,d) and below (a,c) depinning threshold. We consider dumb-bell **(a,b)** and sphero-cylindrical **(c,d)** shells. Nanoparticles act as "virtual" topological defects bearing a *virtual topological charge* $\Delta m_{\mathrm{v}} = 1$.

Next, we treat a qualitatively different case, similar to the one depicted in Figure S1b. For illustration purpose we consider a sphero-cylinder, see Figure S5c, where one NP enforcing $m = 1$ is present. Below the depinning threshold such configuration possesses two $m = 1/2$ defects to fulfil Eq. (10). As in the case above, we limit to

the upper part of the structure, assuming that the lower part is mirror symmetrical. To make the derivation more transparent we set that the spherical patch is represented by $\Delta\zeta_-$ and the cylindrical part by $\Delta\zeta_+$. Consequently, it holds $\Delta m_{\mathrm{K}}(\Delta\zeta_+) = 0$, $\Delta m_{\mathrm{K}}(\Delta\zeta_-) = -1$ (due to Eq. (9)), and $m = 1/2$ *defect* is attracted to the $\Delta\zeta_-$ patch. The *effective topological charge* within patches $\Delta\zeta_-$ ($\Delta\zeta_+$) equals $\Delta m_{\mathrm{eff}}^{(-)} = \Delta m + \Delta m_{\mathrm{v}} + \Delta m_{\mathrm{K}} = 1/2 + 0 - 1 = -1/2$ ($\Delta m_{\mathrm{eff}}^{(+)} = \Delta m + \Delta m_{\mathrm{v}} + \Delta m_{\mathrm{K}} \sim 0 + 1/2 + 0 = 1/2$). Here, we assumed that a half of the central NP bearing $m = 1$ contributes to each half of the sphero-cylinder. To cancel Δm_{eff} in the patches, two pairs $\{$*defect,antidefect*$\}$=$\{m = 1/2, m = -1/2\}$ must be formed. The two *antidefects* are needed to screen the central "virtual" charge enforced by NP. On the other hand, *defects* are moved toward poles. The corresponding structure is "neutral", i.e. represents the ETCC *limit structure* shown in Figure S5d.

---

[1] MacKintosh, F. & Lubensky, T. Orientational order, topology, and vesicle shapes. *Phys. Rev. Lett.* **67**, 1169 (1991).

[2] Helfrich, W. & Prost, J. Intrinsic bending force in anisotropic membranes made of chiral molecules. *Phys. Rev. A* **38**, 3065 (1988).

[3] Lubensky, T. & Prost, J. Orientational order and vesicle shape. *J. Phys. II* **2**, 371–382 (1992).

[4] Fournier, J. Nontopological saddle-splay and curvature instabilities from anisotropic membrane inclusions. *Phys. Rev. Lett.* **76**, 4436 (1996).

[5] Kralj-Iglič, V., Svetina, S. & Žekš, B. Shapes of bilayer vesicles with membrane embedded molecules. *Eur. Biophys. J.* **24**, 311–321 (1996).

[6] Fournier, J. & Galatola, P. Tubular vesicles and effective fourth-order membrane elastic theories. *EPL* **39**, 225 (1997).

[7] Kralj-Iglič, V., Heinrich, V., Svetina, S. & Žekš, B. Free energy of closed membrane with anisotropic inclusions. *EPJ B* **10**, 5–8 (1999).

[8] Fošnarič, M., Iglič, A. & May, S. Influence of rigid inclusions on the bending elasticity of a lipid membrane. *Phys. Rev. E* **74**, 051503 (2006).

[9] Akabori, K. & Santangelo, C. D. Membrane morphology induced by anisotropic proteins. *Phys. Rev. E* **84**, 061909 (2011).

[10] Kralj-Iglič, V., Iglič, A., Hägerstrand, H. & Peterlin, P. Stable tubular microexovesicles of the erythrocyte membrane induced by dimeric amphiphiles. *Phys. Rev. E* **61**, 4230 (2000).

[11] Kralj-Iglič, V., Babnik, B., Gauger, D. R., May, S. & Iglič, A. Quadrupolar ordering of phospholipid molecules in narrow necks of phospholipid vesicles. *J. Stat. Phys* **125**, 727–752 (2006).

[12] Ramakrishnan, N., Ipsen, J. H. & Kumar, P. S. Role of disclinations in determining the morphology of deformable fluid interfaces. *Soft Matter* **8**, 3058–3061 (2012).

[13] Jesenek, D., Perutková, Š., Kralj-Iglič, V., Kralj, S. & Iglič, A. Exocytotic fusion pore stability and topological defects in the membrane with orientational degree of ordering. *Cell Calcium* **52**, 277–282 (2012).

[14] Jesenek, D. *et al.* Vesiculation of biological membrane driven by curvature induced frustrations in membrane orientational ordering. *Int. J. Nanomedicine* **8**, 677–687 (2013).

[15] Perutková, Š., Kralj-Iglič, V., Frank, M. & Iglič, A. Mechanical stability of membrane nanotubular protrusions influenced by attachment of flexible rod-like proteins. *J. Biomech.* **43**, 1612–1617 (2010).

[16] Yu, H. & Schulten, K. Membrane sculpting by f-bar domains studied by molecular dynamics simulations. *PLoS Comput. Biol.* **9**, e1002892 (2013).

[17] Gómez-Llobregat, J., Elías-Wolff, F. & Lindén, M. Anisotropic membrane curvature sensing by amphipathic peptides. *Biophys. J.* **110**, 197–204 (2016).

[18] Mesarec, L., Góźdź, W., Kralj-Iglič, V., Kralj, S. & Iglič, A. Closed membrane shapes with attached bar domains subject to external force of actin filaments. *Colloids Surf., B* **141**, 132–140 (2016).

[19] Perutková, Š. *et al.* Elastic deformations in hexagonal phases studied by small-angle x-ray diffraction and simulations. *Phys. Chem. Chem. Phys.* **13**, 3100–3107 (2011).

[20] Templer, R. H. Thermodynamic and theoretical aspects of cubic mesophases in nature and biological amphiphiles. *Curr. Opin. Colloid Interface Sci.* **3**, 255–263 (1998).

[21] Nelson, D. & Peliti, L. Fluctuations in membranes with crystalline and hexatic order. *J. Phys.* **48**, 1085–1092 (1987).

[22] David, F., Guitter, E. & Peliti, L. Critical properties of fluid membranes with hexatic order. *J. Phys.* **48**, 2059–2066 (1987).

[23] Park, J. M. & Lubensky, T. Sine-gordon field theory for the kosterlitz-thouless transitions on fluctuating membranes. *Phys. Rev. E* **53**, 2665 (1996).

[24] Fournier, J. B. & Galatola, P. Bilayer membranes with 2d-nematic order of the surfactant polar heads. *Braz. J. Phys.* **28**, 329 (1998).

[25] Kamien, R. D. The geometry of soft materials: a primer. *Rev. Mod. Phys.* **74**, 953 (2002).

[26] Selinger, R. L. B., Konya, A., Travesset, A. & Selinger, J. V. Monte carlo studies of the xy model on two-dimensional curved surfaces. *J. Phys. Chem. B* **115**, 13989–13993 (2011).

[27] Napoli, G. & Vergori, L. Surface free energies for nematic shells. *Phys. Rev. E* **85**, 061701 (2012).

[28] Rosso, R., Virga, E. G. & Kralj, S. Parallel transport and defects on nematic shells. *Continuum Mech. Therm.* **24**, 643–664 (2012).

[29] Pairam, E. *et al.* Stable nematic droplets with handles. *Proc. Natl. Acad. Sci. USA* **110**, 9295–9300 (2013).
[30] Iglič, A., Babnik, B., Gimsa, U. & Kralj-Iglič, V. On the role of membrane anisotropy in the beading transition of undulated tubular membrane structures. *J. Phys. A: Math. Gen.* **38**, 8527 (2005).
[31] Kralj-Iglič, V., Remškar, M., Vidmar, G., Fošnarič, M. & Iglič, A. Deviatoric elasticity as a possible physical mechanism explaining collapse of inorganic micro and nanotubes. *Phys. Lett. A* **296**, 151–155 (2002).
[32] Iglič, A. *et al.* Stable shapes of thin anisotropic nano-strips. *Fuller. Nanotub. Car. N.* **13**, 183–192 (2005).
[33] Kralj-Iglič, V. *et al.* Microtubes and nanotubes of a phospholipid bilayer membrane. *J. Phys. A: Math. Gen.* **35**, 1533 (2002).
